# Hydrogen Bonds and Life in the Universe

**DOI:** 10.3390/life8010001

**Published:** 2018-01-03

**Authors:** Giovanni Vladilo, Ali Hassanali

**Affiliations:** 1 INAF-Osservatorio Astronomico di Trieste, Via G.B. Tiepolo 11, 34143 Trieste, Italy; 2 International Center for Theoretical Physics, Strada Costiera, 11, 34151 Trieste, Italy; ahassana@ictp.it

**Keywords:** life in the universe, molecular processes, hydrogen bonding, habitability

## Abstract

The scientific community is allocating more and more resources to space missions and astronomical observations dedicated to the search for life beyond Earth. This experimental endeavor needs to be backed by a theoretical framework aimed at defining universal criteria for the existence of life. With this aim in mind, we have explored which chemical and physical properties should be expected for life possibly different from the terrestrial one, but similarly sustained by genetic and catalytic molecules. We show that functional molecules performing genetic and catalytic tasks must feature a hierarchy of chemical interactions operating in distinct energy bands. Of all known chemical bonds and forces, only hydrogen bonds are able to mediate the directional interactions of lower energy that are needed for the operation of genetic and catalytic tasks. For this reason and because of the unique quantum properties of hydrogen bonding, the functional molecules involved in life processes are predicted to have extensive hydrogen-bonding capabilities. A molecular medium generating a hydrogen-bond network is probably essential to support the activity of the functional molecules. These hydrogen-bond requirements constrain the viability of hypothetical biochemistries alternative to the terrestrial one, provide thermal limits to life molecular processes, and offer a conceptual framework to define a transition from a “covalent-bond stage” to a “hydrogen-bond stage” in prebiotic chemistry.

## 1. Introduction

The search for life beyond Earth relies heavily on our understanding of the only form of biology that we currently know, i.e., the terrestrial one. As a result, the criteria adopted to search for life in remote environments can be affected by Earth-centric bias. To cope with this problem a broad paradigm of life must be defined, wherein terrestrial biology is a special case of a more universal phenomenon [1,2]. The present work aims to make a contribution in this direction in light of the rising interest in the quest for life in the solar system and in extrasolar planets. The recognition that terrestrial-type planets around solar-type stars are relatively frequent [3] is providing new momentum to studies of planetary habitability [4,5,6]. The goal of this paper is to search for physical and chemical constraints for the existence of life that can be used to define new criteria of habitability. Among physical conditions, we are interested in exploring the possible existence of universal limits of temperature for life processes. Temperature variations have a strong impact on biological processes [7,8] and severely affect the capability of an environment to host life. Thermal limits of life can be combined with climate models of terrestrial-type exoplanets to define temperature-dependent criteria of habitability [9,10,11,12,13]. Among chemical conditions, we are interested in exploring the viability of hypothetical biochemistries alternative to the terrestrial one [2] in order to understand whether worlds with exotic conditions may host life or not. For instance, the existence of lakes of liquid hydrocarbons on Titan [14] has raised the interest in the possible existence of life based on non-polar solvents [15,16]. Clearly, the definition of the circumstellar habitable zone [17,18,19] which is essentially based on the liquid water criterion, should incorporate physically-based limits of life, if they exist, and should be revised if alternative biochemistries are plausible.

**Figure 1:**
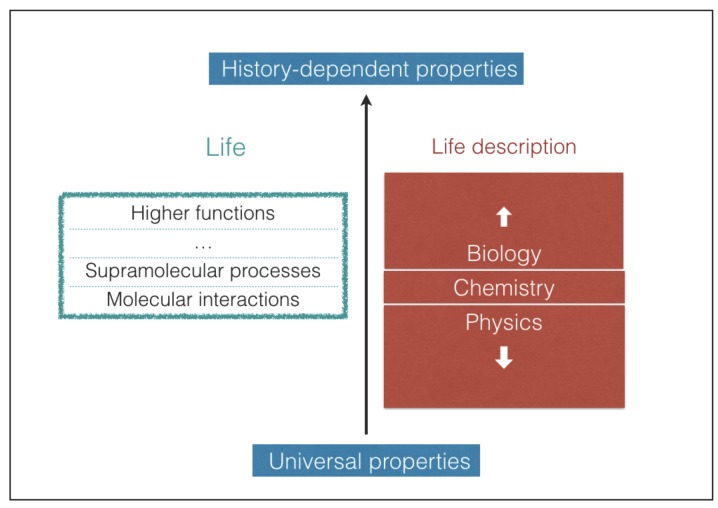
Idealized representation of life as a stratification of levels of increasing functional complexity (green box). Due to the lack of a unified description of life, the different levels are investigated by different scientific disciplines (red boxes). The upper levels are heavily affected by unpredictable events that take place during the evolutionary history of life. The chemical interactions taking place between molecular constituents can be described using the universal laws of physics and chemistry.

Predicting which possible types of biochemistries may take place in the universe is an extremely difficult task [20]. Life embraces different levels of structural and functional complexity, starting from the molecular level up to the highest supramolecular levels, where the most elaborate functions take place (Figure 1). The most complex biological structures and functions result, in large part, from contingent events that shape the pathways of life evolution [21,22]. As a result, the evolutionary outcome is unpredictable and this is true even for molecular structures [23]. Nonetheless, the interactions that take place between biomolecules are mediated by well-known chemical bonds and forces that obey the universal laws of physics and chemistry. In this work we confine our attention to such chemical interactions, which are unaffected by the evolutionary history of life. In this way we can search for common chemical and physical properties of life that can be eventually employed as universal criteria of habitability. Our approach is essentially mechanistic and does not attempt to understand the deep reasons for the emergence of life, which are still hard to grasp despite being the subject of intense research [24]. In the next section we propose a minimum set of requisites for the existence of life driven by molecular processes. In the subsequent sections we use such requisites as a working tool to predict consequences in terms of chemical interactions, the possibility of alternative biochemistries and thermal limits of life processes.

## 2. Requisites for the Existence of Life

Life is usually defined by enunciating a list of properties shared by terrestrial organisms. The list may include metabolism, growth, reproduction, evolution, self-containment and/or other characteristics [25,26,27]. Even if there is no common agreement on which properties should be adopted to define life [28,29,30], we know that every single defining property, originally discovered in the macroscopic world, is sustained by the action of underlying molecular processes. This fact encourages us to adopt a molecular perspective to generalize the concept of life. To keep an open-minded attitude to the nature of hypothetical, non-terrestrial life, we impose a minimal number of requisites that life molecular processes should satisfy. The first requisite that we adopt is:

**Requisite** **1.** Life processes are sustained by functional molecules that perform genetic and catalytic tasks.

Some remarks are necessary to clarify the meaning and implications of this requisite. Genetic tasks aim at exchanging information coded in a sequence of chemical groups. Catalytic tasks aim at reducing energy barriers of chemical reactions used to synthesise or recycle molecular constituents. The possibility of molecular replication, which is considered to be essential for the emergence of life [24], is implicit in Requisite 1 because molecular replication can be provided by genetic and catalytic molecules.

Present-day terrestrial life can be thought of as a particular case that fits Requisite 1, with nucleic acids and proteins playing the roles of genetic and catalytic polymers. Previous evolutionary stages of terrestrial life also fit the requisite since the basic molecular constituents have been preserved in the course of billons years of evolution [21,22]. The same is true for the RNA world [31] because Requisite 1 does not exclude that the same molecule can perform both the genetic and the catalytic tasks.

In addition to present and past terrestrial life, Requisite 1 fits a broad spectrum of hypothetical life forms since it does not specify the nature of the functional molecules. Therefore synthetic biopolymers, such as PNA [32] or others [33], or non-terrestrial biomolecules, if any, may fit the requisite as long as they have genetic and catalytic capabilities. Moreover, it should be clear that the functional molecules may have topologies different from those of terrestrial macromolecules: they do not need to be linear structures and can have any sort of 3D conformation.

Finally, Requisite 1 does not preclude other molecular ingredients from being present in addition to functional molecules. For instance, a molecular medium surrounding the functional molecules (Section 6) and molecular structures providing compartmentalization [34], are expected to be present in any form of life. Any other molecular constituents that do not preclude the operation of the genetic and catalytic functions can also be present. In conclusion, Requisite 1 leaves open the possibility of a broad spectrum of terrestrial or non-terrestrial life forms.

In principle, technological progress could disclose the possibility to build some form of artificial life not based on genetic and catalytic molecules. Artificial life would be virtually impossible to constrain from a physico-chemical point of view and so it would not provide physico-chemical constraints of habitability, which are the final goal of the present investigation. To discard this type of life we introduce an additional condition:

**Requisite** **2.** The molecules with genetic and catalytic capabilities emerge from a prebiotic process of natural selection of molecular constituents.

**Figure 2:**
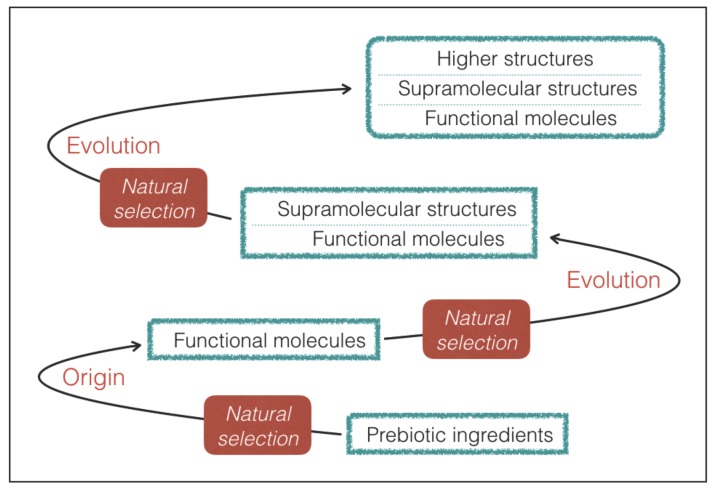
Conceptual framework for the origin and evolution of life adopted in this work. Life is postulated to originate from a prebiotic process of natural selection that leads to the emergence of functional molecules with genetic and catalytic capabilities (Section 2). Subsequent episodes of Darwinian evolution may add structural and functional complexity, always preserving functional molecules as an underlying, essential ingredient of life processes.

In practice, we limit our attention to life that maintains functional molecules at the root of its processes despite the variations driven by Darwinian-type evolution (Figure 2). As we shall see, the requirement of a prebiotic process of natural selection provides a guideline to predict the most likely outcome in terms of molecular properties.

## 3. A Hierarchy of Life Chemical Interactions

Any form of life satisfying Requisites 1 and 2, whether on Earth or not, must share a number of properties. Functional molecules must have long atomic sequences to be able to store a large number of genetic instructions or to acquire a variety of catalytic shapes [35,36]. For simplicity, we call “functional chains” the long atomic sequences of functional molecules, keeping in mind that the topology of such chains does not need to be linear: the chains can form rings or can be branched and form tangled 3D structures. The operation of genetic and catalytic tasks, postulated by Requisite 1, implies that functional chains must interact with each other, exchanging chemical groups between them and with any other molecule involved in such tasks. Here we show that the operation of these molecular processes requires the coexistence of a hierarchy of chemical interactions. We use the word interaction in a broad sense, including in this category both transient encounters between molecular constituents and chemical bonds that hold atomic groups in a more stable fashion. A priori, these interactions may be different from those typical of terrestrial biomolecules. To emphasize this possibility, we introduce an ad hoc nomenclature of interactions (type-1 , type-2 , type-3 ) to classify different types of interactions that are expected to exist in functional molecules.

### 3.1. Types of Interactions

**Figure 3:**
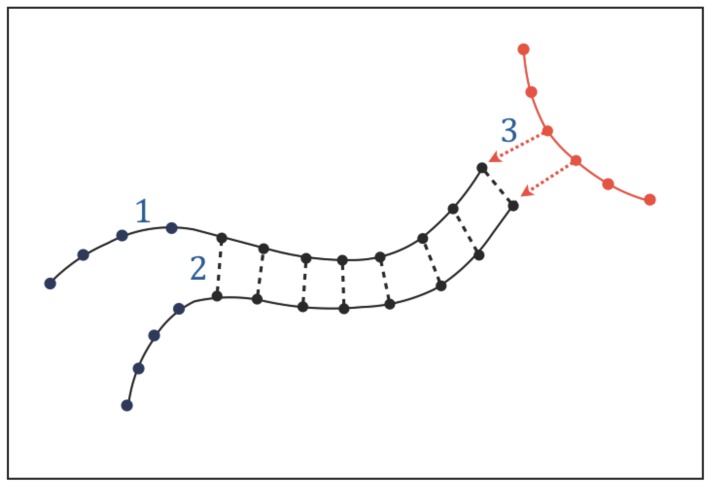
Graphic representation of small portions of functional chains involved in genetic and catalytic tasks. The sketch shows examples of the directional chemical interactions needed for the operation of these tasks. Solid lines: type-1 bonds holding chemical groups (filled circles) along functional chains. Dashed lines: type-2 bonds connecting a sequence of chemical groups along adjacent chains (“chain pairing”). Dotted arrows: type-3 interactions needed for intermolecular recognition. See Section 3.1.

The existence of functional molecules implies that adjacent atoms or chemical groups along their chains must be held together by structural bonds which build their atomic sequences. We call these intra-chain interactions “type-1 bonds” (Figure 3). In addition to intra-chain bonds, the chains of the functional molecules are expected to undergo a particular form of molecular association (or dissociation) that involves a sequential pairing (or unpairing) of a series of chemical groups located along adjacent chains. The existence of this type of molecular association, that we call “chain pairing”, can be predicted by Requisite 1: genetic tasks require copying and translating instructions and these processes in turn require some form of chain pairing (or unpairing); catalytic tasks require the functional molecules to be able to acquire very specific conformations through molecular folding, which is an intramolecular form of pairing. We call the bonds involved in chain pairing “type-2 bonds” (Figure 3).

In addition to intra-chain bonds and chain pairing, the functional molecules will undergo a variety of intermolecular interactions. The need to perform genetic and catalytic tasks implies that part of these interactions must be directional: when the functional molecules interact with each other to synthesise or recycle molecules, exchange information, and catalyze chemical reactions, the interactions must be directional because the molecules involved must recognize each other before performing these tasks. We call the directional interactions needed for intermolecular recognition “type-3 interactions” (Figure 3); these interactions must be pervasive given the essential role of genetic and catalytic tasks.

The main characteristics, biological roles, formation/destruction processes, and requirements of type-1, type-2 and type-3 interactions are summarized in Table 1. The biological roles demonstrate the necessity of such interactions in any form of life based on genetic and catalytic molecules. The requirements will be discussed in the next section. The relationship between type-1, type-2, and type-3 interactions and actual chemical bonds will be discussed in Section 4.

### 3.2. Relative Strengths of the Interactions

In the time interval between their synthesis and disposal, the functional molecules are engaged in the different types of interactions described above. During these interactions, functional chains must maintain their structure to preserve their functionalities. From this general concept we derive quantitative constraints on the relative strengths of type-1 , type-2 , and type-3 interactions.

During chain pairing (or unpairing), some of the type-1 bonds will be exposed to the interactions that establish (or destroy) type-2 bonds. We call $\Delta H_{1}$ and $\Delta H_{2}$ the absolute values of the dissociation energies of type-1 bonds and type-2 bonds, respectively. To preserve the atomic sequence and functionality of the functional chain, the energy exchanged during inter-chain interactions, ≃$\Delta H_{2}$, must be lower than the intra-chain bond energy, $\Delta H_{1}$. This must be true for any type-1 bond of the chain exposed to pairing interactions. Thus, we obtain

(1) $$\max\left\{\Delta H_{2}\right\}<\min{\left\{\Delta H_{1}\right\}}_{2}$$

where ${\left\{\right\}}_{2}$ indicates the subset of bonds of the polymer exposed to chain pairing (or unpairing). In the course of the life-time of functional molecules, some of the type-2 bonds that contribute to the inter-chain stabilization will be exposed to intermolecular interactions. To preserve the conformation and functionality of the chains, the energy exchanged during these intermolecular encounters, $\Delta E_{\mathrm{im}}$, must be lower than the inter-chain bond energy, ≃$\Delta H_{2}$. This must be true for any intermolecular interaction and for any type-2 bond exposed to intermolecular interactions. Thus, we obtain

(2) $$\max\left\{\Delta E_{\mathrm{im}}\right\}<\min{\left\{\Delta H_{2}\right\}}_{\mathrm{im}}$$

where the symbol ${\left\{\right\}}_{\mathrm{im}}$ indicates the subset of bonds exposed to intermolecular interactions. Combining these two relations we obtain

(3) $$\max\left\{\Delta E_{\mathrm{im}}\right\}<\min{\left\{\Delta H_{2}\right\}}_{\mathrm{im}}<\max\left\{\Delta H_{2}\right\}<\min{\left\{\Delta H_{1}\right\}}_{2}.$$

We now assume that intermolecular encounters redistribute the molecular kinetic energy yielding a common mean value of energy for intermolecular interactions, $\langle \Delta E_{\mathrm{im}}\rangle$. Since type-3 interactions are a sub-sample of intermolecular interactions, we have $\langle \Delta E_{3}\rangle ≃\langle \Delta E_{\mathrm{im}}\rangle$. In terms of mean quantities, condition (3) can be rewritten as

(4) $$\langle \Delta E_{\mathrm{im}}\rangle ≃\langle \Delta E_{3}\rangle <\langle \Delta H_{2}\rangle <\langle \Delta H_{1}\rangle \;.$$

The dissociation energies that appear in this inequality are expected to be similar in different interacting molecules because: (i) to optimize the capability of chain pairing, the bond strengths $\Delta H_{2}$ should be similar in different chains; (ii) to optimize the capability of exchanging atoms linked with type-1 bonds, the bond energies $\Delta H_{1}$ should be similar in different chains. Therefore, we argue that the prebiotic process of natural selection (Requisite 2) would lead to the emergence of functional molecules featuring type-1 bonds with similar strengths, as well as type-2 bonds with similar strengths.

**Figure 4:**
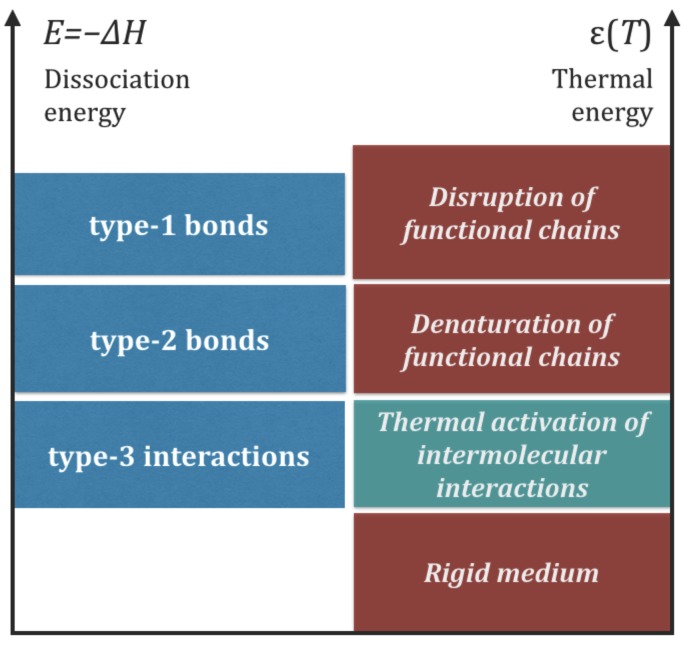
Left side: schematic representation of the hierarchy of chemical of interactions predicted to exist in functional molecules (Section 3). Right side: impact of thermal energy, ϵ(T), on the chemical interactions (Section 7). Upper red boxes: ϵ(T) is too high, leading to the disruption or denaturation of functional molecules. Green box: ϵ(T) is in an optimal range to energize type-3 interactions without disrupting type-2 bonds. Lowest red box: ϵ(T) is too low to energize intermolecular interactions.

In summary, the strengths of chemical bonds and the intermolecular energy exchanges in life functional molecules lie in three distinct energy bands: an upper band characteristic of type-1 bonds, an intermediate band characteristic of type-2 bonds, and a lower band characteristic of intermolecular interactions, including type-3 interactions. This hierarchy of interactions must be present in any form of life that satisfies Requisites 1 and 2 because no assumptions have been made on the type of molecules involved in the processes. The relationships (4) are sketched in the left side of Figure 4. The right side of the same figure illustrates the impact of thermal collisions in different intervals of thermal energy, ϵ(T), which will be discussed in Section 7.

## 4. Chemical Bonds Suitable for Life Molecular Interactions

To make a practical use of condition (4) we now discuss which chemical interactions have the capability to play the role of type-1 , type-2 and type-3 interactions. To set the problem in a universal perspective, we need to be sure that the properties of chemical bonds are the same everywhere in the universe. Astronomical spectroscopic observations suggest that this is the case. For instance, the spectral lines observed in remote sources can be fitted with the physical parameters of atomic/molecular transitions measured in terrestrial laboratories. The most accurate spectroscopic data of remote intergalactic clouds do not show evidence of variation of the fine-structure constant up to a look-back time of ∼12 Gyr [37]. The fine-structure constant governs the strength of electromagnetic forces and its constancy implies that atomic energy levels do not vary in space-time. As a result, the repertoire of chemical bonds and forces is the same on Earth as in remote worlds. This repertoire features strong bonds, such as covalent, ionic and metallic bonds, and weaker interactions, such as van der Waals forces, hydrogen bonds and halogen bonds. The physical nature of such interactions can be traced back to quantum mechanical, electrostatic and/or polarization effects. The main properties of the most frequent chemical interactions in terrestrial biology are summarized in Table 2, Table 3 and Table 4. In the rest of this Section we discuss the potential capability of all known chemical interactions, including those that are absent or uncommon in terrestrial life, to play the role of type-1, type-2, and type-3 interactions.

### 4.1. Intra-Chain Structural Bonds (Type-1 Bonds)

Condition (4) requires type-1 bonds to have the highest values of dissociation energy in functional molecules. In addition, in order to build up functional chains with specific geometries, type-1 bonds should be directional. The strongest chemical bonds in nature are covalent, ionic, and metallic bonds. In covalent bonding adjacent atoms share their electrons in common orbitals; since the geometry of the orbitals creates optimum bond angles, covalent bonding is very directional. Ionic bonding arises from the electrostatic attraction between ions that have exchanged one or more electrons; since there are no shared electron pairs to repel each other, ionic bonds tend to be less directional than covalent bonds. Metallic bonding arises from the electrostatic attractive force between free conduction electrons and a lattice of positively charged metal ions; as a consequence of electron delocalization, metallic bonds lack directionality. Both ionic bonding and metallic bonding tend to form regular lattice structures.

The poor (or lack of) directionality and the regular lattice structures typical of ionic and metallic bonding are not appropriate for storing information, as required by genetic molecules, or creating complex conformations, as required by catalytic molecules. These characteristics are instead provided by covalent bonding. Based on the above arguments and supported by the example of terrestrial biomolecules, we argue that the prebiotic process of natural selection postulated by Requisite 2 would lead to the emergence of functional chains with type-1 bonds of covalent type. Some contribution of ionic or metallic bonds in the chains would not alter the conclusions of this work, which are focused on type-2 bonds and type-3 interactions, rather than type-1 bonds, as we discuss below.

### 4.2. Inter-Chain Pairing Interactions (Type-2 Bonds)

Condition (4) requires type-2 bonds to have dissociation energies lower than type-1 bonds. Since covalent bonds span a broad range of strengths (Table 2), the weakest covalent bonds could in principle be used as type-2 bonds and the strongest ones as type-1 bonds. In practice, however, covalent bonds cannot be used in chain pairing because covalent bonding would establish new electronic orbitals, altering the atomic sequences of the chains, contrary to the requirement that the functional molecules should not be affected by inter-chain pairing (Section 3.2). Clearly, type-2 bonds should have a “non-invasive” character in addition to being weaker than covalent bonds. These requirements can only be satisfied by non-covalent bonds, namely van der Waals forces, hydrogen bonds, and halogen bonds.

Van der Waals (vdW) forces are weak interactions (≲1 kcal/mol) that take place between molecular dipoles or multipoles (Table 3). With a few partial exceptions, such as Keesom forces, most vdW forces are isotropic. Due to their lack of directionality, vdW interactions cannot play the role of type-2 bonds despite being weak and non-invasive. Nonetheless, they do contribute to the structural stability of the molecules since they are present in any type of molecular interaction. In particular, their contribution is relevant if the functional molecules are packed by stronger bonds, such as hydrogen bonds, that freeze intermolecular distances in a range where vdW forces are attractive.

**Figure 5:**
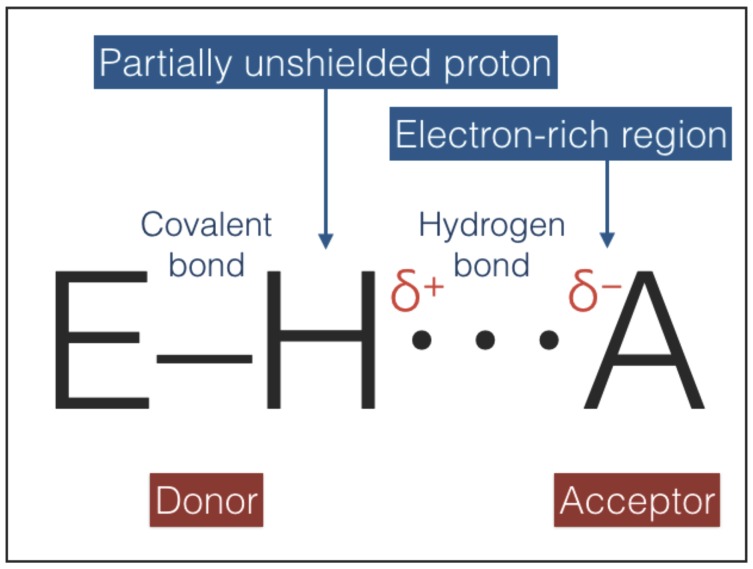
A hydrogen bond, sketched in this figure, is an attractive interaction between a hydrogen atom from a molecule or a molecular fragment E–H in which E is more electronegative than H, and an atom or a group of atoms, A, in the same or a different molecule [39]. Due to the difference in electronegativity, the proton in the hydrogen nucleus is partially unshielded and the group E–H, called the donor, is polarized. The group A, called the acceptor, must possess at least one electron-rich region (e.g., a lone-pair-possessing atom or a π system).

The hydrogen bonding interaction, sketched in Figure 5, combines an electrostatic force, induced by a partially unshielded proton, with variable contributions of covalent bonding and vdW forces [39,40,41,42,43]. The strengths of hydrogen bonds range between ≈1 and ≈40 kcal/mol (Table 4). Hydrogen bonds are directional and the directionality increases with strength because the stronger bonds are more difficult to distort: an increase of strength is accompanied by a shortening of the equilibrium distances and a deepening of the potential wells; deeper wells have a higher curvature at the minimum and oppose larger forces to distorsions [44]. Hydrogen bonds can play the role of type-2 interactions because they are directional, weaker than covalent bonds, and non-invasive: most of the time hydrogen bonds are interactions between two molecules that retain their individuality [43].

**Figure 6:**
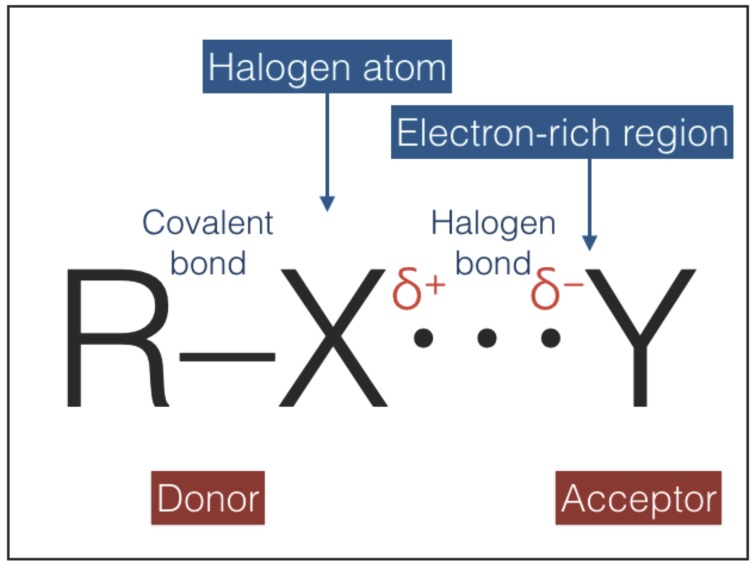
A halogen bond, sketched in this figure, is an attractive interaction between an electrophilic region associated with a halogen atom in a molecular entity and a nucleophilic region in another, or the same, molecular entity [46,47,48]. The halogen atom, X, is covalently bonded to the atom/chemical group R. The donor R–X must have an electrophilic region on its electrostatic potential surface. The acceptor Y must possess at least one nucleophilic region (e.g., a lone-pair-possessing atom or a π system).

Halogen bonds (Figure 6) are primarily electrostatic, but with polarization, charge-transfer and dispersion contributions all playing an important role [46,47,48]. Halogen bonds are very directional, more than hydrogen bonds, with the acceptor Y approaching X along the extension of the R–X bond. The strength of halogen bonds is comparable to that of hydrogen bonds and decreases with increasing electronegativity of X (i.e., in the order I > Br > Cl > F). In principle, halogen bonds can play the role of type-2 interactions due to their similarities with hydrogen bonds (i.e., directionality, weakness, and non-invasive character). However, halogen atoms have significantly larger radii than hydrogen atoms [49] and therefore halogen bonding is more sensitive to steric hindrance than hydrogen bonding [48]. This difference may impact negatively on the possibility of chain pairing since steric hindrance between adjacent groups can restrict torsional bond angles. Another aspect that disfavors halogen bonds is the very low cosmic abundances of halogens: the abundances of these elements relative to hydrogen lie in the range between $10^{-6.5}$, in the case of Cl, down to $10^{-10.5}$, in the case of I [50]. As a result, halogen bonding may become as important as hydrogen bonding only in astronomical environments with a significant local enhancement of halogens relative to the average cosmic abundance.

In conclusion, type-2 interactions for chain pairing (Figure 3) can only be established via hydrogen bonding, with some potential contribution of halogen bonding. Even though some particular molecular structures, such as leucine zippers [51], can provide complementary surfaces that do not involve hydrogen or halogen bonds, such structures would not be suitable for chain pairing.

### 4.3. Interactions for Intermolecular Recognition (Type-3 Interactions)

Condition (4) shows that the intermolecular forces must be the weakest of life molecular processes. In this respect, vdW forces can be important since they are the weakest chemical interactions. However, vdW forces are not suitable for intermolecular recognition via type-3 interactions because they lack directionality and because they decay more quickly with intermolecular distance, *r*, with a typical decay law $r^{-6}$ (Table 3). The fast decay limits the possibility of identifying a molecular partner unless the two molecules are brought together to a very low *r*. In contrast, hydrogen bonds are well suited for recognition because they are directional and because their decay with *r* is more gradual (Table 2). Thanks to this gradual decline, hydrogen bonds have an orienting effect, useful for molecular recognition, also at a relatively large *r*, when the two molecules start to approach each other [44]. Halogen bonding may contribute to type-3 interactions, despite the low cosmic abundance of halogens, as long as its decay with *r* is gradual as in the case of hydrogen bonding [52]. It should be stressed that vdW forces are also important for intermolecular recognition, but at much shorter length scale. Even though some particular polymers with sequence-specific polarizabilities may attain molecular recognition by vdW interactions [53], intermolecular recognition via type-3 interactions requires hydrogen (or perhaps halogen) bonding.

### 4.4. The Unique Role of Hydrogen Bonding

The above discussion indicates that among all types of known chemical interactions, only hydrogen (and possibly halogen) bonds can play the role of type-2 bonds or type-3 interactions. These interactions should be sufficiently versatile to provide the range of strengths required to satisfy condition (4): type-2 , structural bonds need to be relatively strong, whereas type-3 interactions used in intermolecular recognition must be relatively weak. This versatility is present in hydrogen and halogen bonding since the strength of these bonds can vary depending on the chemistry of the molecular groups forming the interaction as well as the medium through which they occur. The strength of halogen bonds is tunable with a change of the halogen element X since the R–X donor ability changes in the order I > Br > Cl > F [48]. Hydrogen bonds are known to be extremely versatile: with a proper change of molecular environment, even the same configuration E–H⋯A (Figure 5) can provide a spectrum of strengths and lengths [41,42,43]. The strength is also fine-tuned by solvent/dielectric screening: a dielectric medium will weaken the electrostatic interactions according to the value of its dielectric constant, $\epsilon_{r}$ (see Table 5). Further versatility is provided by a transient breaking of hydrogen bonds without complete detachment of the molecules: this type of breaking requires energies lower than the dissociation energy because it leaves the molecules essentially in the same position. For instance, a transient breaking of a hydrogen bond in liquid water requires 1.5 kcal/mol [54], whereas the binding energy of the water dimer is ΔH(375) = −3.2 kcal/mol [55].

In assessing the relative importance of hydrogen and halogen bonding, it is important to keep in mind that the molecular interactions in biological systems need to be soft to facilitate fluctuations in the structure and hence to make them dynamical systems. Hydrogen bonding can provide structure fluctuations because it is sensitive to nuclear quantum effects [56,57] owing to the low mass and non-classical nature of the proton. Quantum effects are expected to be much less important in halogen bonding owing to the high masses of halogen atoms, which are between 19 times (F) and 127 times (I) the proton mass.

An important property related to nuclear quantum effects concerns the transfers of protons or of H atoms which may take place through hydrogen bonding [43]. It is hard to emphasize the importance of proton transfer, which is at the heart of acid-base chemistry reactions [58,59,60]. Also electron transfer, a key aspect of life molecular processes, is coupled in different ways to proton transfer [61]: this coupling is necessary to maintain electrical neutrality in biological systems. Proton transfer is also central to the dynamical equilibrium that determines the pH of water [62]. Proton transfer is not provided by halogen bonding and, concerning this aspect, hydrogen bonding is unique among any other chemical interaction.

Another interesting feature of nuclear quantum effects has to do with the sensitivity of proton delocalization to the length of the hydrogen bond. For example, by compressing the oxygen-oxygen distance between water molecules from 2.8 to 2.4 Å one can induce/enhance the delocalization of the proton along the hydrogen bond and give it a more covalent character [63]. This example shows that the pressure of the medium in which molecules are embedded may play an important role since pressure variations may shift the length H⋯A (Figure 5). In this regard, hydrogen bonds are quite versatile in that small perturbations to the heavy atom distances can sensitively affect the extent of proton delocalization. This has an effect of increasing/decreasing their strength.

Within the context of the origins of life, another important factor that should be considered is the role of hydrogen bonding in the photophysics and photochemistry of small organic molecules. High energy irradiation during the conditions where pre-biotic chemistry emerged is likely to have facilitated chemistry on both the ground and excited electronic states. Hydrogen bonding in this case also plays an important role since the extent of proton delocalization along hydrogen bonds can affect the energies at which photons are absorbed [64,65,66], and also the fate of molecules and products formed on the excited state [67,68].

In conclusion, hydrogen bonding is an essential ingredient for both type-2 and type-3 interactions and, at variance with halogen bonding, it is also a crucial player in a variety of quantum-based molecular processes that provide charge transfer and lay the foundation for building the fluctuating, dynamical systems that characterize life. Based on the above arguments, we argue that the prebiotic processes that lead to natural selection of genetic and catalytic molecules (Requisite 2) will inevitably lead to the emergence of functional molecules with a significant ability of hydrogen bonding. The example of terrestrial life is in line with these expectations: in terrestrial biology hydrogen bonds play an overwhelming role in genetic and catalytic molecules [42,44], whereas halogen bonds only play a limited role in some supramolecular assemblies [48,69]. Halogen bonds in supramolecular structures of terrestrial life probably represent an outcome of a later evolutionary step that has taken place without altering the main features of the underlying network of functional molecules emerged in the early stages of evolution (Figure 2). Finally, it is worth noting that the hydrogen-bond strengths and lengths lie at the confluence of energy and length scales of a variety of thermal, chemical, mechanical, and electrostatic processes [70]. This remarkable accordance gives further support to the importance of hydrogen bonding, which occupies a privileged position in terms of strength and length scale.

## 5. Viable Chemistries of Life

**Figure 7:**
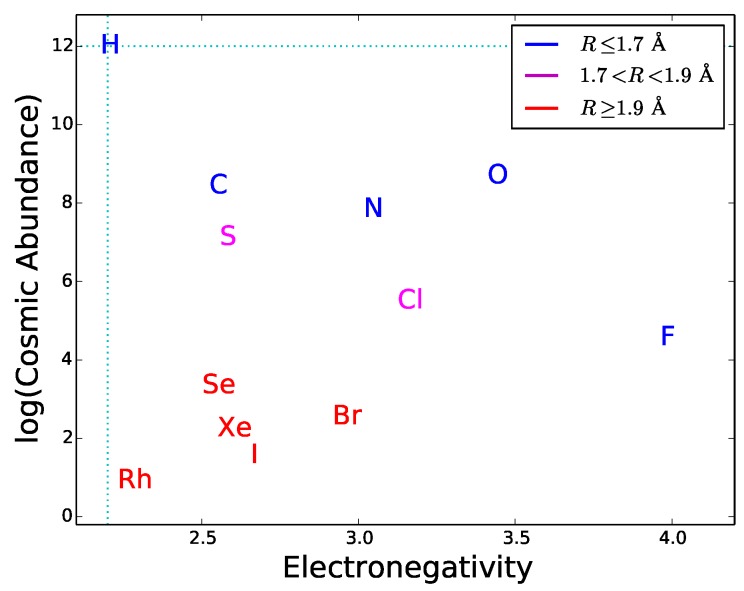
Cosmic abundance by number [50] versus electronegativity [72] of chemical elements more electronegative than hydrogen; van der Waals radii of the elements [49] are color-coded as indicated in the legend. Local concentrations ore depletions of elemental abundances may yield significant deviations relative to the cosmic abundances displayed in the figure.

Since most type-2 and type-3 interactions need to be mediated by hydrogen bonds, functional molecules must have active sites with hydrogen bond donors and/or acceptors. This requirement can be used as a guideline to explore the potential of different elements to play a role in hypothetical biochemistries alternative to the terrestrial one. By definition of hydrogen bond, the element E in the donor group E–H has to be more electronegative than H. This restricts the choice of E since only a small number of elements have this property. The atomic radius also restricts the choice because the electric dipole effect, an important component of hydrogen bonding, becomes weaker with increasing atomic radius. Finally, the cosmic abundance of E gives a general indication of the possibility for a given element to be incorporated in hydrogen bond donors; this indication should be used with caution since local concentrations or depletions in astronomical environments may significantly alter the average cosmic abundances. In Figure 7 the electronegativity is plotted versus radius and cosmic abundance for the elements more electronegative than H. One can see that only a handful of chemical elements is suitable for hydrogen bonding. Half of these elements are very uncommon in the universe and have relatively large atomic radii. The best suited and cosmically abundant are C, N, and O. The donor groups N–H and O–H provide hydrogen bonds with typical strengths of ∼6–8 kcal/mol, whereas C–H donors are characterized by weak hydrogen bonding [44]. Another relatively abundant, electronegative element is sulphur. The dissociation energy of S-based hydrogen bonds is extremely low [71], possibly due to the relative large size of the sulphur atom. Due to their weakness, C- and S-based hydrogen bonds are less directional than N- and O-based hydrogen bonds. Besides being important in donor groups, N and O can also act as hydrogen bond acceptors thanks to the presence of lone pairs of electrons in their outer orbitals (Figure 5). The fact that N, and O are extensively used in the active chemical groups of terrestrial biomolecules is arguably related to the capability of these atoms to establish strong and directional hydrogen bonds, acting both as acceptors and in donor groups. For these reasons, we expect that molecules with N-based and O-based hydrogen bonding would commonly emerge in any form of life (not only the terrestrial one), as a result of the prebiotic process of natural selection of molecular constituents (Requisite 2).

The hydrogen-bonding requirements can also be used to predict if elements not used in terrestrial biochemistry could be employed in exotic forms of life. As an example, we consider silicon, which is sometimes considered a candidate for alternative biochemistries. Silicon does not have hydrogen bonding capability because its electronegativity (1.9) is lower than that of hydrogen. Therefore silicon is not suitable for participating in type-2 bonds and type-3 interactions. Arguably, this is the reason why silicon is absent in the active sites of terrestrial nucleic acids and proteins despite its large abundance in the Earth’s crust. Quite interestingly, terrestrial life does employ silicon to build up rigid structures of plants and diatoms [26]. This proves that life does have the capability of extracting and incorporating silicon from the non-biological world, but does not employ this element for the active sites of functional molecules. The silicon-based structures of terrestrial life are a possible example of biological features emerged from evolution without altering the underlying, dominant molecular ingredients (Figure 2).

## 6. The Molecular Medium of Life

Life processes would not be able to take place without the aid of molecules that support the activity of the genetic and catalytic molecules. In particular, these functional molecules would benefit from the structural support provided by a surrounding molecular medium. In general, the functional molecules will be synthesized in one site and perform their tasks in a different location. Therefore, the molecular medium should be able to: (i) allow the functional molecules to move from the site of their assemblage to the site of their activity and (ii) keep the functional molecules in a specific site during their activity. To provide mobility to the functional molecules, the molecules of the medium should easily detach from each other: they should form a fluid substrate rather than a rigid matrix. At variance with the functional molecules, a small size is optimal for the molecules of the medium because: (i) a small size helps filling and supporting the 3D contour of functional molecules whatever the many conformations they may acquire; (ii) a small size facilitates the mobility of the functional molecules: in the absence of a filling medium of small molecules, the long functional molecules would be packed together limiting the free movement of each other. A medium of small molecules is also important to redistribute the thermal energy among functional molecules (Section 7). For all the above reasons we argue that, at the stage of life origin, the emergence of functional molecules (Requisite 2) will be accompanied by the parallel emergence of a liquid molecular medium.

The intermolecular interactions between the molecules of the medium must be weak: only weak bonds can easily break, in a transient fashion, to allow the mobility of functional molecules through the medium. Weak chemical bonding can be provided by vdW forces and hydrogen bonds. Depending on which type of weak interaction is dominant, the molecular medium might play different roles in hypothetical forms of non-terrean biochemistry. At the bottom level, the medium could just be a “filler” that occupies the intermolecular space providing mobility to the embedded functional molecules. This would happen if vdW forces were the only type of interactions between the molecules of the medium. In this case, the medium would not feature hydrogen-bond-based type-3 interactions. To play a more active role, mediating type-3 interactions with functional chains, the molecules of the medium should be able to establish hydrogen bonds. If the molecules of the medium have both a donor and an acceptor, the medium can form a network of hydrogen bonds. A network of this type leads to the emergence of collective properties which can be exploited by life processes, such as proton transfers (Section 4.4), cooperative effects, reactivity to electric fields, and the (so-called) “hydrophobic” effect. The cooperativity arises because the ability of donor and acceptor groups to form hydrogen bonds is further increased by an increase in polarity when the bonds form a collective ensemble [42]. The reactivity to electric fields arises because a hydrogen bond network is, in practice, an ensemble of dipoles that can be collectively re-oriented, providing extra capabilities of transmission of electric signals through the medium. The hydrophobic effect arises, in large part, from entropy variations [73], but it also requires a molecular medium with directional interactions. The hydrophobic effect is extremely important to provide and maintain the 3D structures of functional molecules [43].

From the above discussion it is clear that the hydrogen-bonding capability of different molecules can be used to predict which of them are the most suited to form the molecular medium of hypothetical, non-terrestrial biochemistries. In practice, we can classify the molecules according to their numbers of hydrogen-bond donors, $N_{D}$, and acceptors, $N_{A}$. Examples of cosmically abundant, small molecules classified in this way are shown in Table 5.

The first example is CH${}_{4}$, which has 4 weak donors but no acceptors. In a medium of pure methane, the only type of directional interaction would be a weak hydrogen bond between a C–H donor and an acceptor (but not a donor) of a functional chain. In these conditions, most interactions between methane and functional chains would be mediated by non-specific vdW forces. A further limitation is that CH${}_{4}$ molecules are not be able to form a hydrogen-bond network. Therefore, the capability of methane to actively organize functional chains would be very limited. These constraints cast doubts on the viability of a biochemistry with methane as the liquid medium. For instance, molecular chains should find their own way to fold in reasonable times without the aid of the hydrophobic effect. Whether a biochemistry of this type is viable or not remains to be demonstrated.

The second example is NH${}_{3}$, which has 3 donors and 1 acceptor. As a result, ammonia can interact with both an acceptor and a donor of a functional molecule. In addition, NH${}_{3}$ molecules can form a network of hydrogen bonds. However, since NH${}_{3}$ has only one acceptor, ammonia molecules can only establish a single hydrogen bond between them, forming a sort of 1D hydrogen-bond network. As a result, in a medium of pure ammonia, a hydrogen bond connection between a NH${}_{3}$ molecule and a functional molecule would interrupt the hydrogen-bond network. These constraints would limit the cooperativity, polarization and hydrophobic effects in hypothetical biochemistries based on liquid ammonia.

Finally, H${}_{2}$O has 2 donors and 2 acceptors. The dual hydrogen-bond donor-acceptor functionality is a unique property of the H${}_{2}$O molecule which explains most of the special properties of water [42]. Water molecules can form a 3D hydrogen bond network where each molecule can establish up to four hydrogen bonds with neighbouring molecules. Thanks to this fact, water molecules can establish directional links with the functional molecules while still being hydrogen-bonded with other water molecules (i.e., without interrupting the hydrogen-bond network). In terrestrial life, water molecules are involved in the stabilization of intermolecular complexes [74] and can mediate the molecular recognition of chemical groups (e.g., Figure 10.7 in [43]).

Clearly, the list of small molecules displayed in Table 5 is not exhaustive. Another potential candidate is H${}_{2}$S, since sulphur is relatively abundant and provides fairly weak but noticeable hydrogen bonds [71]. Even molecules with a larger number of atoms can be particularly interesting from the point of view of the hydrogen-bonding ability. A remarkable example, also shown in Table 5, is formamide, with triple donors and acceptors. Not surpringly, this molecule has an excellent ability of hydrogen bonding [75]. An exhaustive study of potential candidates for the molecular medium of life is beyond the purpose of the present work. The above examples clearly indicate that hydrogen-bond criteria provide a useful guideline for predicting suitable candidates.

### Length Scales of Life Molecular Interactions

The length scales of intermolecular interactions provide further arguments to assess which molecules are best fit to form the molecular medium of life processes. In particular, we consider the Bjerrum length, which is the distance at which the Coulomb energy between two elementary charges *e* is comparable in magnitude to the thermal energy, $k_{B}T$. In a medium with relative dielectric constant $\epsilon_{r}$ the Bjerrum length is

(5) $$\lambda_{B}=\frac{e^{2}}{4\pi \epsilon_{\circ }\epsilon_{r}k_{B}T}$$

where $\epsilon_{\circ }$ is the vacuum permittivity [38]. This length can be used to discriminate ranges of intermolecular distances, *r*, where electrostatic interactions dominate over thermal energy ($r\ll \lambda_{B}$) from ranges where thermal energy dominates ($r\gg \lambda_{B}$). In Table 5 we show representative values of $\epsilon_{r}$ and $\lambda_{B}$ for small, cosmically abundant molecules in liquid phase. One can see that $\lambda_{B}$ changes dramatically in different molecular media, from ≃100 nm in the case of liquid methane, down to ≃0.7 nm for liquid water. For comparison, the typical length scales of intermolecular interactions are in the range $r_{\mathrm{HB}}≃$ 0.15–0.3 nm for hydrogen bonds [44], and $r_{\mathrm{vdW}}≃0.3$–0.6 nm for vdW interactions. This comparison shows that liquid water is unique since $\lambda_{B}$ and $r_{\mathrm{vdW}}$ have similar length scales: the thermal energy is comparable to the intermolecular electrostatic energy between charged molecules. As a result, in liquid water the thermal energy can be used to dissolve or provide mobility to charged biomolecules. This is not true in liquid ammonia or liquid methane, where $r\ll \lambda_{B}$. In these liquids the electrostatic forces acting between charged biomolecules are much stronger than the thermal energy and, as a result, these intermolecular interactions are “frozen”. These arguments cast further doubts on the viability of a biochemistry based on liquid ammonia or, even worse, liquid methane.

An important aspect associated with the Bjerrum length in water is the two-length scale hydrophobic effect that was postulated by Chandler [76]. According to Chandler’s theory of hydrophobic solvation, the work required to create a cavity in water grows as the volume for short length-scales, while it grows as the surface area at long length scales. The critical crossover length for idealized spherical hydrophobic solutes occurs at ∼1 nm, which is close both to the Bjerrum length in water as well as the length of vdW interactions. This remarkable convergence of length scales is a characteristic property of water.

## 7. Thermal Limits of Hydrogen-Bond Life

Thermal motions provide a source of kinetic energy at the molecular scale. This energy can drive conformational changes, dissolution of the weakest chemical bonds, and diffusion of the functional molecules. Terrestrial life shows remarkable examples of these and other possible ways of harvesting thermal energy, such as the molecular motors, which are able to convert thermal fluctuations into a directed force [70,77]. The Brownian motion of the molecules of the medium redistributes the thermal energy to the functional molecules involved in life processes. We assume that the thermal energy is essential to drive intermolecular interactions and, in particular, type-3 interactions. This may sound like a strong assumption but on the other hand, in absence of thermal energy redistribution, an *ad hoc*, pervasive energy source internal to the medium should be invoked to energize the huge number of intermolecular interactions needed in life processes.

Due to energy redistribution, we have $\langle \Delta E_{\mathrm{im}}\rangle ≃\langle \Delta E_{3}\rangle ≃\langle \epsilon \left(T\right)\rangle$, where $\langle \epsilon \left(T\right)\rangle ≃n\;k_{B}T$ is the mean thermal energy at temperature *T*. The impact of the thermal energy on hydrogen bonds can be used to infer thermal limits of life: only above a proper temperature threshold the energy is sufficient to drive intermolecular interactions; on the other hand, if the temperature is too high, the type-2 bonds that hold paired chains will be disrupted (see Figure 4). These constraints provide the basis for a methodology, that we propose here, to infer thermal limits of life. The methodology can be applied not only to terrestrial life, but also to any other form of life based on genetic and catalytic molecules. As we show below, the application of our methodology requires to know the dissociation energies of hydrogen bonds in a variety of molecular structures. A limit of this approach is that accurate experimental data of this type are hard to obtain, in particular for weak hydrogen bonds of dynamical molecular structures [42,44]. Notwithstanding, to show the viability of this approach, we provide preliminary estimates based on well-known types of hydrogen bonds. To introduce the methodology we discuss the temperature dependence of thermal destruction rates of type-2 and type-3 interactions.

### 7.1. Strength of Chemical Bonds and Reaction Rates

**Figure 8:**
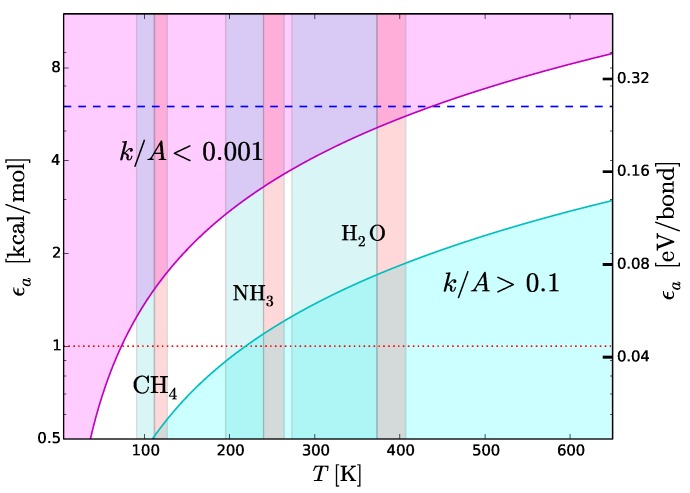
Activation energy of chemical reactions, $\epsilon_{a}$, plotted as a function of temperature, *T*, and normalized reaction rate, k/A. Chemical bonds with strengths comparable to $\epsilon_{a}$ are more quickly disrupted in the cyan region and are stable in the magenta region. Light blue vertical strips: liquid-phase temperature limits for pure methane, ammonia and water, with boiling points calculated at pressure *p* = 1 bar; light red vertical strips: interval of boiling points for pure methane, ammonia and water calculated between *p* = 1 bar and *p* = 3 bar. Red dotted line: approximate lower boundary of weak hydrogen bonds (Table 4). Blue dashed line: minimum strength of hydrogen bonds critical for inter-chain stability. See Section 3.2 and Section 7.1.

Type-2 bonds and type-3 interactions have opposite requirements of stability in presence of thermal collisions. To minimize the probability that a type-2 bond with structural functions is damaged by an exchange of kinetic energy, the rate of potentially harmful thermal interactions must be very low. On the other hand, the rate of type-3 interactions must be extremely fast in molecular recognition and transportation. Recognition needs to be very fast because the only way a molecule can recognize a potential partner is by checking many possible associations with other molecules [42]. Also the interactions between the functional molecules and the molecules of the medium must “switch on” and “off” very rapidly to allow the movement of the functional molecules through the medium. To discriminate slow and fast processes we calculate the reaction rates, *k*, as a function of temperature, *T*, with the Arrhenius relation

(6) $$k=A\exp\{-\epsilon_{a}/RT\}$$

where $\epsilon_{a}$ is the activation energy, *A* the number of collisions of reactants per unit time and *R* the universal constant of gas. In molecular recognition the activation energy is the interaction energy of type-3 interactions, i.e., $\epsilon_{a}≃\Delta E_{3}≃\Delta E_{\mathrm{im}}$. The fastest possible rate is attained when the reactions take place as soon as the molecules collide, i.e., when k/A≃1. This ideal condition, called the “diffusion controlled” regime, can be approached at low $\epsilon_{a}$ and high *T*. The cyan region in Figure 8 shows the region of the plane *T*-$\log\epsilon_{a}$ where k/A>0.1. This region is optimal for fast intermolecular interactions such as type-3 interactions. Let us now consider structural type-2 bonds. Their thermal disruption rate, that in this case we wish to minimize, can be calculated taking $\epsilon_{a}≃\Delta H_{2}$. To minimize the disruption, the normalized reaction rate should be k/A≪1. The magenta region in Figure 8 shows the region of the plane *T*-$\log\epsilon_{a}$ where k/A<0.001. This region is optimal for the preservation of type-2 bonds in presence of thermal collisions. Figure 8 reproduces the scheme skecthed in Figure 4 in a quantitative way, emphasizing its dependence on *T*. We use this quantitative scheme to infer thermal limits for life processes.

### 7.2. Upper Thermal Limit

To derive an upper temperature limit we require the thermal energy not to exceed the dissociation energy of type-2 bonds. In principle, the most stringent limit are provided by weak hydrogen bonds. Among cosmically abundant elements, the weakest hydrogen bonds are based on C and S (Section 5). However, the weakest hydrogen bonds cannot be the dominant form of type-2 bonds, otherwise it would not be possible to find even weaker hydrogen bonds required for type-3 interactions (see inequality (4)). This argument suggests that type-2 bonds must be composed, in large part, by stronger hydrogen bonds such as those based on N and O (Section 5). Weaker bonds based on C or S could be present, but only to play a secondary role. The example of terrestrial biomolecules is in line with these predictions: the dominant types of inter-chain bonds in proteins and nucleic acids are N–H⋯O bonds, while carbohydrates are characterized by O–H⋯O bonds [42]. Detailed studies of many examples of biomolecules show that weak C–H⋯O bonds are quite frequent in proteins, nucleic acids and carbohydrates, but only to play a secondary role for adjacent, stronger N–H⋯O and O–H⋯O bonds [44]. Accordingly, the inter-chain stability is basically determined by N–H⋯O and O–H⋯O bonds. Thus, given the absence of other chemical elements that can enter in the donor or acceptor groups (Section 5 and Figure 7), we expect N- and O-based hydrogen bonds to be the most common ones in any type of functional molecules and not just in the terrestrial ones. Both types of hydrogen bonds are strong, with binding energies in the range between 6 and 8 kcal/mol. The tighter temperature limits are provided by the bonds with N–H donor, which are the weakest ones in this category. The typical strength of these bonds is 6 kcal/mol, a value that we adopt to estimate an upper thermal limit (dashed line in Figure 8). To convert this energy into a temperature limit we use Equation (6). By taking $\epsilon_{a}$= 6 kcal/mol and imposing a reaction rate sufficiently low to prevent disruption of inter-chain bonds (k/A<0.001), we obtain $T≲4.4\times 10^{2}$ K. An hypothetical biochemistry taking place at temperatures slightly higher than this limit, could still make use of O-based hydrogen bonds, but not of N-based hydrogen bonds. At even higher temperatures, F-based hydrogen bonds could still resist because of their very high values of dissociation energy (Table 4) resulting from the high electronegativity of fluorine (Figure 7). However, the disruption of C-, N- and O-based hydrogen bonds at such high temperatures and the low cosmic abundance of F would dramatically restrict the viability of a biochemistry based (only) on fluorine hydrogen bonds.

### 7.3. Lower Thermal Limit

A lower thermal limit for life processes can be derived requiring the temperature to be sufficiently high to keep the molecular medium in the liquid phase. This type of lower limit is unaffected by variations of pressure because the melting point is basically constant, at variance with the boiling point which increases with pressure. Examples of liquid-phase temperature intervals for CH${}_{4}$, NH${}_{3}$ and H${}_{2}$O are shown in Figure 8. The disadvantage of this approach is that the resulting temperature limits are not universal since they depend on the “choice” of the molecular medium. To bypass this limitation, we derive a lower temperature limit by requiring the thermal energy to be sufficiently high to power intermolecular recognition. Since type-3 interactions used in molecular recognition need hydrogen bonds, the intermolecular bonds must have energies above the minimum hydrogen-bond energy ≃1 kcal/mol (dotted line in Figure 8), i.e., $\epsilon_{a}≃\Delta E_{3}≳1$ kcal/mol. Since we want the activation/deactivation of hydrogen bonds to be fast, the reaction rate must approach the diffusion controlled regime, i.e., k/A>0.1. Inserting the limits $\epsilon_{a}≳1$ kcal/mol and k/A>0.1 in Equation (6), we obtain $T≳2.2\times 10^{2}$ K. This limit partly overlaps with the liquid-ammonia temperature range (Figure 8), supporting the possibility that ammonia could be, to some extent, a viable medium for life processes (Section 6). On the other hand, the limit $T≳2.2\times 10^{2}$ K is largely above the boiling point of methane (Figure 8). In other words, in the temperature range of liquid methane, the thermal energy would be insufficient to activate type-3 interactions. In these conditions, an additional source of energy able to trigger such interactions would be required. This fact casts further doubts on the viability of methane as a medium for life processes (Section 6). Given the uncertainties in estimating the minimum strength of hydrogen bonds [44,78], the limit $T≳2.2\times 10^{2}$ K should be considered as tentative.

### 7.4. Comparison with the Thermal Limits of Terrestrial Life

If our methodology is correct, the approximate limits $2.2\times 10^{2}$ K $≲T≲4.4\times 10^{2}$ K, derived using hydrogen-bond criteria, should be valid for any form of life based on genetic and catalytic molecules. To test the validity of the methodology, we compare such limits with the thermal limits of terrestrial life. We confine our attention to life in which the functional molecules are in an active state rather in survival conditions, i.e., life with active metabolism and capability of reproduction. For this type of life, terrestrial extremophiles provide the limits $2.5\times 10^{2}$ K $≲T≲4.0\times 10^{2}$ K [8]. Therefore, the limits based on hydrogen bonding are somewhat broader than, but consistent with those of terrestrial life. To properly interpret this result, it is important to recall that the hydrogen-bond limits are relevant at the molecular level. Structures of supramolecular complexity, which must be present even in the simplest cells, may introduce additional thermal constraints, narrowing the temperature interval suitable for life processes. Indeed, this seems to be the case in terrestrial life, where the interval of thermal tolerance tends to narrow with increasing organism complexity [8,12]. Taking into account the narrowing of the temperature interval at supramolecular level, we can conclude that the predictions for hydrogen-bond life are in good agreement with those of terrestrial life.

## 8. Conclusions

We have shown that molecular life processes sustained by functional molecules with genetic and catalytic capabilities must display a hierarchy of chemical interactions operating in three distinct energy bands. Among all known chemical interactions, only hydrogen bonds can mediate the low-energy, directional interactions required for the operation of genetic and catalytic tasks. Therefore the functional molecules, and the molecular constituents that interact with them, must have extensive capabilities of hydrogen bonding. This conclusion is reinforced by the fact that the quantum effects peculiar of hydrogen bonding are needed to build a dynamical system of fluctuating conformations, typical of life. Since life based on genetic and catalytic molecules represents a broad generalization of terrestrial life, we may conclude that hydrogen bonding represents a universal language of life molecular interactions.

The hydrogen-bonding requisites of functional molecules provide an original perspective to test the viability of hypothetical, non-terrestrial forms of biochemistry. Moreover, the capability of forming hydrogen bonds explains, among other things, why O, N and C are present (and Si is absent) in active sites at the surface of functional molecules. The capability of interacting with the functional molecules via hydrogen-bonding indicates which molecules are best suited to form the molecular medium of life. Among small, cosmically abundant molecules, water, ammonia and methane have decreasing capability of supporting the operations of functional molecules via hydrogen bonding. The lack of hydrogen-bond acceptors in the CH${}_{4}$ molecule casts doubts on the viability of a biochemistry based on liquid methane. Another drawback of liquid methane is that its thermal energy is insufficient to drive the hydrogen-bond interactions required for fast intermolecular recognition.

The hydrogen-bond requirements provide thermal limits that are valid for any active form of biochemistry based on molecules with genetic and catalytic properties. The stability of interchain structures mediated by N-based hydrogen bonds yields an approximate upper limit $T≲4.4\times 10^{2}$ K. The need for fast hydrogen-bond interactions driven by thermal energy provides an approximate lower limit $T≳2.2\times 10^{2}$ K. This limit is uncertain due to the difficulty of estimating dissociation energies of weak hydrogen bonds. Accurate estimates of dissociation energies may help to derive tighter thermal limits in the future. To this end the database of hydrogen bond properties should be extended to include a large number of molecular structures potentially relevant for life processes. Studies of this type will benefit from the application of new techniques aimed at characterizing hydrogen bonds [79].

The properties of hydrogen bonds should be investigated as a function of the pressure of the medium since the length and strength of the bonds is influenced by pressure variations. In this way, a connection could be established between the ambient pressure of astronomical environments and the capability of such environments to host life molecular processes. In principle, this type of work could be applied to the definition of the habitable zone for planets with different levels of surface pressure [10].

The findings of our study can be applied to any form of life originated from a prebiotic process of natural selection of genetic and catalytic molecules. Given the necessity of hydrogen bonds, molecules with hydrogen bonding capabilities are expected to emerge in the final steps of prebiotic chemistry (Figure 2). Perhaps it is no coincidence that formamide, one of the most successful molecular precursors of prebiotic chemistry [80], has an excellent capability of hydrogen bonding. Interestingly, studies of the molecular evolution leading to the RNA world have identified molecular constituents where hydrogen bonding plays an essential role [81]. The hydrogen bond perspective provides a conceptual framework to define two stages of prebiotic chemistry: a “covalent-bond stage”, that can develop in a wide variety of astronomical environments (interstellar clouds, protoplanetary disks, minor bodies, planets, etc.), and a “hydrogen-bond stage”, that can only develop in a limited fraction of environments where the physical and chemical conditions permit the establishment of a network of hydrogen bonds. The RNA world and the emergence of life can only take place in the hydrogen-bond stage. Physical and chemical conditions conducive to hydrogen bonding should be used as a general criterion to search for habitable environments in the universe.

## Figures and Tables

**Table 1:** Classification of chemical interactions according to the role played in functional molecules.

Interaction	Type 1	Type 2	Type 3
Description	Bonds betweennearby atoms alongfunctional chains	Bonds involved in theassociation of portionsof functional chains	Transient interactionsbetween external sitesof functional molecules
Nature	Intra-chain	Inter-chain (intramolecularor intermolecular)	Intermolecular
Biological role	Structural	Encoding of instructions—Folding of catalytic molecules	Intermolecular recognition
Formation/destruction	Synthesis/disposalof functional molecules	Chain pairing/unpairing	Approach/removal of functional molecules
Requirements	Resilient to type-2 andtype-3 interactions	Resilient to type-3 interactions—Non-invasive (to preserve type-1 bonds)	Non-invasive (to preservetype-1 and type-2 bonds)

**Table 2:** Chemical bonds and interactions that play a major role in life molecular processes.

Forces/Chemical Bonds	Covalent Bonds	Hydrogen Bonds (HB)	van der Waals (vdW)
Physical nature	Quantum mechanical	Mixed (see Table 4)	Electrostatic/polarization
Dissociation energy (−kcal/mol)	≃ 40–250	≃ 1–40	≃ 0.5–a few
Directionality	Directional	Directional	Nearly isotropic
Decay with distance ${}^{a}$	Complicated, short range	Roughly ${}^{b}$∼$r^{-2}$	Typically ∼ $r^{-6}$ (see Table 3)

${}^{a}$ Approximate dependence of the interaction energy on intermolecular distance, *r* [38]. ${}^{b}$ Ions like NH${}_{3}^{+}$ and COO${}^{-}$ can form salt bridges where the effective interaction scales as $r^{-1}$; these ions can interact with water which forms an effective charge-dipole interaction scaling as $r^{-3}$; the weakest hydrogen bond is the dipole-dipole interaction such as between water molecules which scales as $r^{-6}$.

**Table 3:** Main types of van der Waals forces.

	Keesom Forces	Debye Forces	London Forces
Physical nature	Dipole−dipole	Dipole−induced dipole	Fluctuating dipoles
Partners	Permanent dipoles	Polar molecule & any other molecule	Any pair of molecules
Attraction	Attractive or repulsive	Always attractive	Always attractive
Decay with distance ${}^{a}$	∼$r^{-3}$ (fixed dipoles)	∼$r^{-6}$ (fixed dipole)	∼$r^{-6}$
	∼$r^{-6}$ (freely rotating dipoles)	∼$r^{-6}$ (freely rotating dipole)	

${}^{a}$ Approximate dependence of the interaction energy of two molecules as a function of intermolecular distance, *r* [38].

**Table 4:** Main properties of Hydrogen Bonds [44,45]. The definitions of weak and strong bonds may change from author to author.

	Weak	Strong	Very Strong
Electrostatics/polarization contribution	Moderate	Dominant	Significant
Quantum-mechanical contribution	Vanishing	Weak	Pronounced
Dissociation energy (−kcal/mol)	≈1–4	≃4–15	≃15–40
Examples	C–H⋯O	O–H⋯O=C	[F⋯H⋯F] ${}^{-}$
	C–H⋯N	N–H⋯O=C	[N⋯H⋯N]${}^{+}$
	O–H⋯Π	O–H⋯O–H	

**Table 5:** Hydrogen bond (HB) and other properties of cosmically abundant, small molecules.

	HB Strength & Directionality	$N_{D}{\;}^{a}$	$N_{A}{\;}^{b}$	Potential HB Partners	HB Network	$\epsilon_{r}\left(T_{\ell }\right){\;}^{c}$	$\lambda_{B}$ (nm)
Methane	Weak	4	0	Only acceptors	Absent	1.7	98.5
Ammonia	Strong	3	1	Acceptors & donors	Present (1D)	25	3.42
Water	Strong	2	2	Acceptors & donors	Present (3D)	78	0.72
Formamide	Strong	3	3	Acceptors & donors	Present (3D)	109	0.52

${}^{a}$ Number of donor hydrogen atoms in each molecule. ${}^{b}$ Number of lone pairs of electrons in the outer shells of C, N or O. ${}^{c}$ Value of the relative dielectric constant at a temperature $T_{\ell }$ in the liquid-phase interval: $T_{\ell }=$ 100 K, 195 K, 298 K and 293 K for methane, ammonia, water and formamide, respectively.
